# Environmental Bond Degradation of Different Laminated Glass Panels

**DOI:** 10.3390/polym16142040

**Published:** 2024-07-17

**Authors:** Alaa El-Sisi, Mohamed Elsawi Mahmoud, Hesham El-Emam, Ahmed Elbelbisi, Hani Salim

**Affiliations:** 1 Department of Civil Engineering, Southern Illinois University, Edwardsville, IL 62026, USA; 2 Department of Civil and Environmental Engineering, University of Missouri, Columbia, MO 65211, USA; moetqb@missouri.edu (M.E.M.); elemamh@missouri.edu (H.E.-E.); salimh@missouri.edu (H.S.)

**Keywords:** laminated glass, environmental effect, bond tests, degradation

## Abstract

Since buildings are designed to endure over time, it is crucial to comprehend how laminated glass (LG) windows, and consequently, the polymer interlayer materials, respond to weathering. This paper explores the impact of accelerated humidity on the mechanical properties of several polymer interlayer materials and LG sections. The study specifically focuses on three polymer interlayer materials of industrial interest: polyvinyl butyral (PVB), ethylene-vinyl acetate (EVA), and ionomer (SG). To examine the environmental effects, testing setups were devised to subject the polymeric materials and LG panels to specific conditions. Uniaxial tension coupons and LG disks were submerged in a water bath to simulate the environmental effect. A dedicated testing fixture was designed and manufactured for the LG disks. The results showed that the properties of EVA, including strength, maximum strain, and toughness, were not significantly affected by the environmental conditions. However, the properties of SG5000 were notably impacted, with a significant reduction in its bond strength due to water immersion.

## 1. Introduction

The mechanical properties of polymer interlayers are greatly affected by weathering, which can alter their behavior due to temperature, humidity, and solar radiation. Numerous studies have highlighted the notable impact of weathering on the physical and mechanical characteristics of polyvinyl butyral (PVB), the interlayer most commonly used in laminated glass [1,2,3]. For instance, in a study by Saad et al., they examined PVB’s response to ultraviolet (UV) radiation and found that it undergoes significant changes, with cross-linking being the predominant effect during UV irradiation [4].

In a study conducted by Andreozzi et al., they explored the impact of humidity, thermal cycles, and UV radiation on PVB [5]. Their findings revealed that humidity had a more significant effect on adhesion compared to its influence on the bulk response of the material. Furthermore, exposure to UV radiation caused a remarkable stiffening of the PVB, leading to a reduction in adhesion. This adhesion is crucial for the interlayer’s ability to contain glass shards in the event of a window break. After conditioning the specimens in all three cases, Andreozzi et al. employed an oscillatory test to measure the rheological properties of the PVB [5]. However, it is essential to note that using tensile and bond tests, as conducted in the study presented in this paper, may yield fresh and potentially different conclusions from oscillatory tests.

Several authors have investigated the degradation mechanisms of both EVA and SG materials. For instance, Serafinavicius et al. conducted an experiment using glass beams that were laminated with SG, PVB, and EVA [6]. They exposed these beams to a combination of high temperature, humidity, and UV radiation within a controlled climatic chamber, where the humidity was maintained at 50%. The researchers performed long-term four-point bending tests and observed that the aging effect caused by humidity had minimal influence on the materials. Moreover, they found that the combined effect of temperature, humidity, and UV radiation had a similar impact on the materials as UV radiation alone.

Delincé et al. examined the impact of artificial weathering, specifically humidity and UV radiation, on the shear-bond characteristics of PVB and SentryGlas^®^ Plus (SGP) [7]. They employed various types of mechanical tests to compare the localized effects from shear tests with the overall effects from bending tests on laminated glass plates. In all cases, a visual assessment was conducted to detect signs of delamination, and for the samples, the light transmittance was measured both before and after exposure to environmental effects. Notably, no defects were observed according to the standard evaluation criteria for the tested samples. The key takeaway from the study is that mechanical tests can serve as valuable tools for assessing the effects of weathering on shear-bond properties, complementing the visual evaluation prescribed in standards and facilitating the calibration of design values based on statistical analysis.

Butchart et al. presented the findings of their experimental peeling tests, which aimed to study adhesion levels under varying moisture conditions [8]. In these tests, specimens of PVB were sandwiched between a layer of glass and a layer of foil backing. The results revealed a significant decrease in adhesion between the glass and interlayer when water was present, with the adhesion being less than half of what was observed in dry conditions.

Weller et al. conducted various aging experiments on modified PVB, thermoplastic polyurethane (TPU), ionomer (SG), and EVA to assess their long-term stability [9]. The aging scenarios included temperature storage tests, climatic stress tests, and tests under aggressive media and high irradiation, all using small-scale test specimens. The aging tests had a significant impact on both the appearance and material properties of the interlayer materials.

Based on the results, it was concluded that SG and TPU demonstrated the best performance after undergoing the different aging tests. These materials proved to be highly suitable for use as laminated glass interlayers in both indoor and outdoor applications, offering excellent long-term durability.

Ensslen conducted a comprehensive study investigating the behavior of laminated glass specimens when subjected to various weathering conditions [10]. The experiments included subjecting some specimens to UV radiation in a solarium, subjecting others to cycles of temperature and humidity degradation, and simply exposing some specimens to outdoor weathering for two years in different climates. To compare the artificially weathered specimens with those exposed to outdoor weathering, monotonic shear tests were performed. The experimental analysis revealed that moisture penetration of the PVB interlayer at the edges of the glass had a detrimental effect on the durability of laminated glass. This resulted in a decrease in shear stiffness and bond strength. On the other hand, the aging of the interlayer caused by UV radiation and high air temperatures led to stiffening of the material properties. However, the study showed that this stiffening did not compromise the structural safety of the laminated glass, depending on the duration and intensity of the exposure.

Antolinc carried out a three-point bending test on laminated glass under elevated temperature conditions within an environmental chamber [11]. The specimens used in the test consisted of two fully tempered glass plates bonded together with interlayers of EVA and PVB. The tests were conducted at specific temperatures of 23 °C, 35 °C, and 60 °C, with measurements taken after the specimens reached these designated temperatures. The study revealed that laminated glass with the EVA interlayer exhibited more favorable behavior overall when exposed to elevated temperatures, compared to specimens with the PVB interlayer. However, the EVA interlayer did show a limitation, as it began to tear at the temperature of 60 °C. To further explore the bending behavior of laminated glass, the researchers recommended conducting additional tests with smaller temperature intervals, including temperatures below room temperature and even sub-zero temperatures.

Martín et al. performed tests at high strain rates on seven distinct polymer interlayers, which comprised three different types of PVB products, one SG product, two EVA products, and a TPU product, all examined at three different strain rates [12]. The researchers compared the mechanical and optical properties of the unaged specimens with those exposed to thermal cycles, high temperatures, and moisture. The findings showed that the unaged specimens of PVB and SG exhibited the highest stiffness, while EVA demonstrated the highest ductility. Additionally, PVB and SG had the highest tensile strength among the tested materials. Furthermore, EVA and TPU displayed a higher resilience to the effects of aging factors and strain rates when compared to the other interlayers.

Numerous studies have focused on investigating the mechanical properties of interlayer materials under both static and dynamic strain rates. Some of these works include those by [13,14,15,16,17,18,19,20,21,22,23,24,25,26,27,28]. Among these studies, the most commonly used polymer material in safety laminated glass is PVB. PVB’s mechanical response is highly dependent on time, and it has the ability to elongate to several times its initial length. A study highlighted the significant improvements in performance and durability of civil engineering structures through fiber-reinforced polymers (FRPs) and fiber-reinforced concrete (FRC) [29]. FRPs like carbon FRP, glass FRP, and basalt FRP enhanced strength-to-mass ratios and were effective in post-earthquake repairs with notable durability and fatigue resistance. Additionally, integrating short fibers into concrete improved crack resistance and tensile strength. However, challenges remained in optimizing fiber states under load and addressing corrosion in alkaline environments. Flax/glass hybrid composite laminates in marine environments showed a 90% improvement in flexural strength and a 128% increase in modulus compared to pure-flax laminates [30]. These properties were lower than pure-glass laminates, but hybrid laminates exhibited intermediate water uptake, balancing mechanical properties, durability, and environmental impact. Lastly, laminated glass reinforced with PVB interlayers demonstrated a significantly higher ultimate bearing capacity under wheel load conditions, averaging 42.0 kN compared to monolithic glass at 11.3 kN [31]. It maintained structural integrity even after cracking and prevented dangerous fragment dispersion upon failure. Researchers are not only interested in investigating the properties of polymers [32,33,34,35,36,37,38] and beneficial use in civil applications [29,30,31], but there is also a growing interest in integrating smart materials, used in multiple civil applications [39,40], to improve the performance of polymers [41,42].

Test methods for checking on the durability of LSG regarding UV radiation, moisture, and air temperature are generally dealt with in the international standard DIN EN ISO 12543-4 [43]. That standard is partly used by PVB interlayer manufacturers for their regular quality controls with respect to chemical, physical, and optical characteristics of LSG, especially the PVB interlayer. In addition, nowadays, the standard gets increased attention focusing on test procedures required for the CE-marking of LSG in compliance with EN 14449 [44], obligatory for all members of the European Community beginning in March 2007. Within the scope of manufacturers’ quality control tests of LSG samples after accelerated weathering in the laboratory according to reference [43], or weathering under natural climatic conditions, are subjected to various, partly non-standardized test methods (e.g., compression/shear test, Pummel test, and bake-and-cook test). Basic test goals include assessing the adhesion character and possible delamination between interlayer and glass and analyzing the shear strength. Of further interest are UV transmission and moisture concentration of the PVB interlayer. A visual inspection regarding color changes (e.g., yellowing) is also performed. Quantifying an important mechanical parameter, such as the shear stiffness, using the shear modulus (as a function of time), after the long-term influence of enhanced climatic aging is missing within these quality control programs. Also, no statement on the load-carrying behavior of weathered large-scale architectural LSG is made. Therefore, the main part of this publication is the investigation of these open questions. Transferring the test results of weathering samples into practice is performed by numerical parameter studies with the finite-element method (FEM).

## 2. Experimental Study

**Figure 1:**
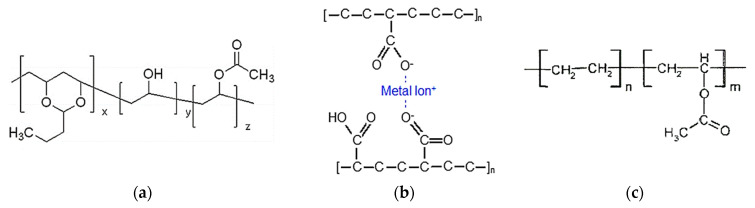
Chemical structure: (**a**) PVB, (**b**) SG, and (**c**) EVA.

Various interlayer materials are readily accessible in the market, and glass processors frequently employ them to create laminated glass systems. Manufacturers typically receive these materials in sheet rolls with specified thicknesses. The materials include PVB, SG, and EVA, having the chemical structure shown in Figure 1. The properties of the polymer sheets used in this particular study are provided in Table 1.

In order to achieve proper adhesion between the polymer interlayer material and the glass, a lamination protocol is employed. During this lamination process, certain changes may occur in the material properties of the interlayer material compared to its original state when received from the manufacturer. This phenomenon typically arises due to the specific lamination procedure used to bond the interlayer material to the glass system. To evaluate and study these effects, the lamination procedure will be carried out on the test specimens. The lamination will be conducted following the process recommended by the manufacturer, which can vary depending on the type of material and the manufacturer involved.

Four different laminated glass panels were procured in dimensions of 12 in × 12 in. The laminated glass panels are composed of two 6.3 mm (0.25 in) glass layers and one 1.5 mm (0.06 in) polymer layer. The polymer interlayer types are PVB, EVA, and SG. For PVB and SG laminates, annealed glass layers were used; however, for EVA laminate, both annealed and tempered glass layers were used. The glass panels with PVB and SG interlayers were evaluated by OLDCASTLE, Orlando, FL, USA/glass fabricator, while EVA panels were procured from MOAG, Georgetown, IN, USA.

### 2.1. Environmental Effects

The environmental investigation in this paper involves accelerated humidity weathering through water immersions. This section provides a detailed explanation of the procedures used to apply these effects on the samples.

**Figure 2:**
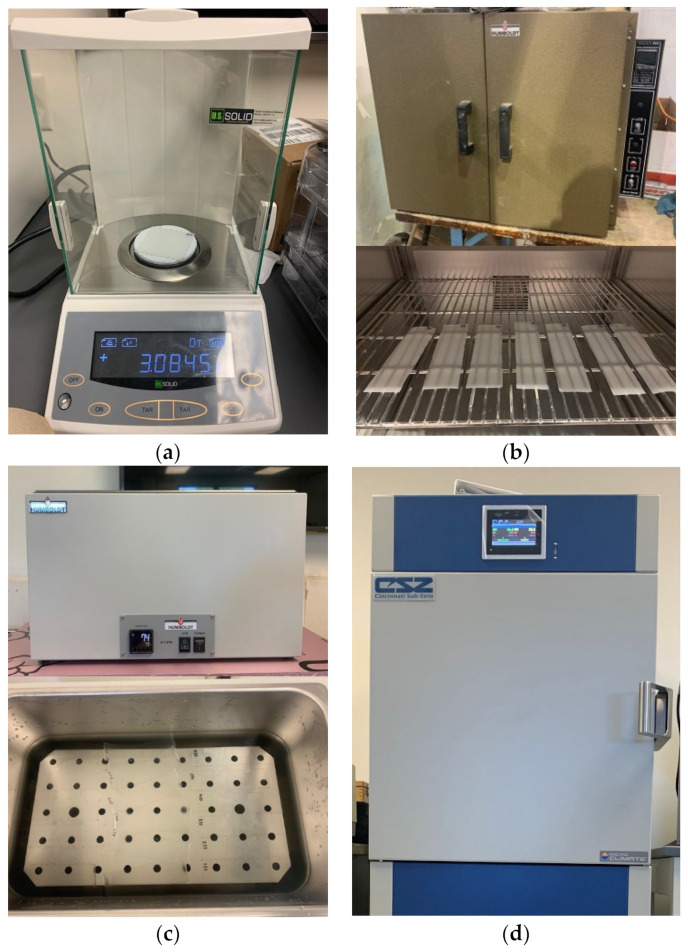
Accelerated-weathering equipment; (**a**) scale, (**b**) water immersion bath, (**c**) drying oven, and (**d**) CSZ environmental chamber.

To assess the water immersion effect on interlayer sheets, the water absorption for each material is determined following the standards ASTM D570-98 and ISO 62 [45,46]. Initially, the interlayer sheets are cut into 5 × 1.5-inch rectangular strips. These specimens are then weighed using a scale with a precision of up to 0.1 mg (Figure 2a) before being placed in a thermostatic water bath (Figure 2b). The water bath is filled with distilled water, maintaining a temperature of 73.4 ± 1.8 °F, until the water level reaches approximately one inch above the specimens (Figure 2b). At specific intervals of 1, 2, 4, 8, 16, 24, 48, 96, and 168 h, the specimens are removed from the water bath and re-weighed to the nearest 0.1 mg, following the procedure used by [21]. After 168 h, the specimens undergo drying in an oven at 40 °C for 24 h. They are then re-weighed (Figure 2a,c) to determine the total moisture content and verify the impact of water absorption on the interlayer sheets.

After cutting the 36 laminated glass disks by the water jet, they were dried by using the oven for 24 h at 40 °C, and the dry weight was reported. Twelve samples were tested in the dry state (DS) while the other 24 samples were placed in the water bath. After 168 h, the specimens were taken from the water bath and re-weighed after drying the surface. For the 24 samples, 12 samples were tested immediately in the saturated state (SS). The other 12 samples were dried in an oven at 40 °C for 24 h and then re-weighed to verify total moisture content and tested in the dry state (SDS).

### 2.2. Sample Preparation

After curing the sheet using the lamination procedure, a uniaxial tension test was performed for cured materials to identify the material characteristics with and without the water submersion effect. Cured/processed sheets were tested in 1.5 mm (0.06 in) thicknesses for PVB and EVA sheets and 0.9 mm (0.035 in) thicknesses for SG5000 sheets. 

**Figure 3:**
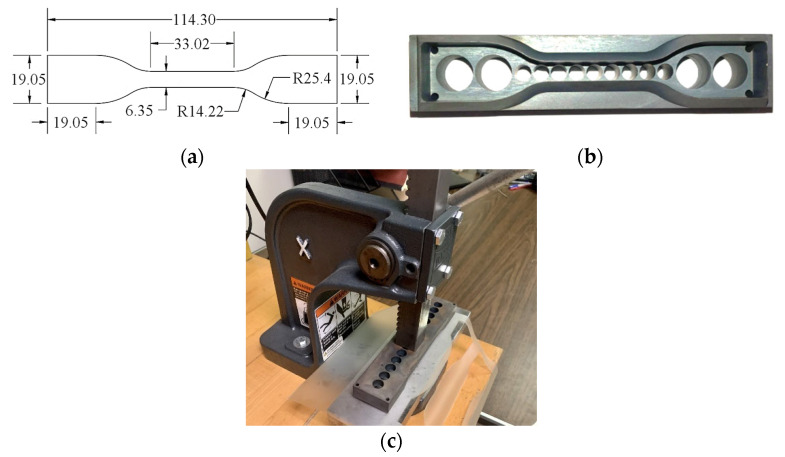
Specimen preparation: (**a**) static specimen geometry, (**b**) static cutting die, and (**c**) specimen stamping. (Dimensions in mm).

The static testing sample employed a standard Type IV specimen geometry, as illustrated in Figure 3a, in accordance with ASTM D638-10 [35]. To ensure precise specimen dimensions, a steel cutting die was fabricated, as seen in Figure 3b,c. Before the testing process, the 25.4 mm (1-inch) central gauge length, Lg, was marked with fine black lines or points using a permanent marker pen, enabling strain monitoring during the test with the assistance of a high-speed camera. The thickness and width of the test section were measured at three different locations using digital calipers, providing accuracy up to 0.0127 mm (0.0005 in).

**Figure 4:**
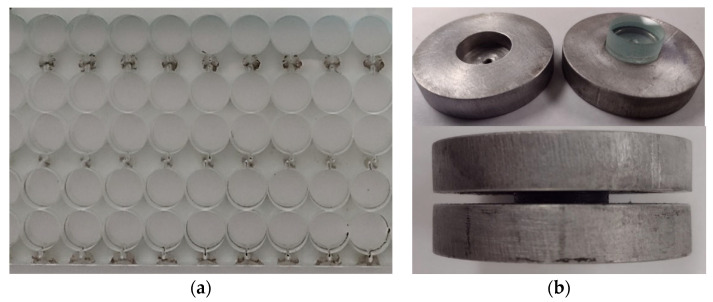
Disk specimen preparation: (**a**) cutting 0.75 in disks from 12 in × 12 in panels and (**b**) aluminum disks. (Dimensions in mm).

For the bond test, the 12 in × 12 in panels were cut into 19.0 mm (0.75 in) circular disks using a Water Jet CNC machine, Figure 4a. A total of 36 disk samples were cut for testing considering three repetitions for every test case as seen in Table 2. To protect the glass from failure due to the stress concentration caused by the fixture, the glass layers are mounted to aluminum disk enclosers before the testing, by using high-strength quick-curing epoxy, as seen in Figure 4b.

### 2.3. Test Setup

In this study, experimental tests were performed on both the interlayer materials and the laminated glass disks with and without the environmental effect. In this section, the testing setup and details will be explained. The environmental effect in this study is water absorption. The experimentation will be carried out in two distinct stages. In the initial stage, a specific portion of the material will undergo exposure to environmental effects. Subsequently, in the second stage, both samples with and without environmental effects will be tested to assess and compare their mechanical properties.

**Figure 5:**
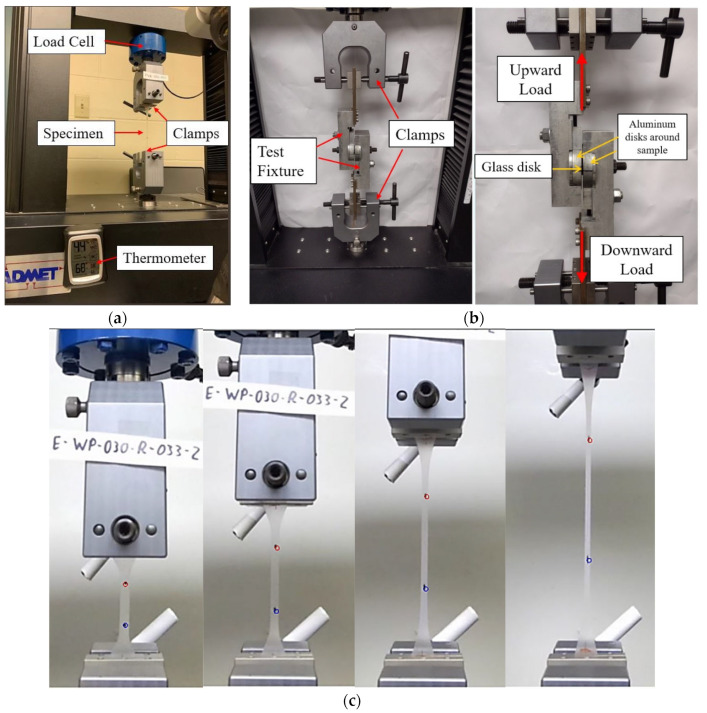
Mechanical testing; (**a**) uniaxial testing of interlayer coupon, (**b**) bond test of laminated glass disks, and (**c**) measuring strain using PhotoTrack V2021 software.

All tests were performed in a temperature-controlled environment at room temperature. An electromechanical static testing frame was used, as seen in Figure 5. The apparatus used in this study has a total travel distance of 18 inches. This device is equipped with precise load cells and a linear variable differential transformer (LVDT) to accurately measure the specimen’s total extension. Additionally, there is a data acquisition system integrated with the apparatus, allowing the test data to be efficiently transferred to computer software for further analysis. In addition, the total distance between the two grips is also recorded. The deformation of the gage length of the specimen was calculated using a high-resolution camera.

The device was used to run both the material characterization uniaxial tension tests of the interlayer and the bond/shear test of laminated glass disks. The interlayer coupon shown in Figure 2 was marked and attached to the machine grips directly as seen in Figure 5a. For the bond/shear test, a testing fixture was designed and manufactured to apply the shear stress on the laminate glass samples up to failure. The main idea of this test fixture was taken from previous works. The testing device contains two similarly built aluminum pieces, in which the aluminum enclosure that contains the laminated glass shear sample was clamped on both sides between adjustable aluminum pieces, see Figure 5b. This kind of fastening minimizes the moments generated due to the eccentric load introduction inside the glass sample. Consequently, a pure shear loading of the interlayer was guaranteed. The tensile load at the ends of the fixture was applied by a static machine previously described. 

During the testing process, the computer continuously records load data from a 2-kip load cell at a precise interval of 0.01 s. Simultaneously, a camera captures the entire event, and later, the footage is transformed into pictures. The software PhotoTrack is then employed to determine the relative displacement between the two dots, as depicted in Figure 5c. With the obtained load–displacement data, a stress–strain diagram can be constructed, providing a valuable tool for comparing material behavior throughout this paper.

## 3. Results and Discussion

In this section, the results of the study will be presented including the uniaxial tension test of polymer coupons and direct shear tests of laminated glass disks with and without water immersion environmental effects.

### 3.1. Tensile Test Results

**Figure 6:**
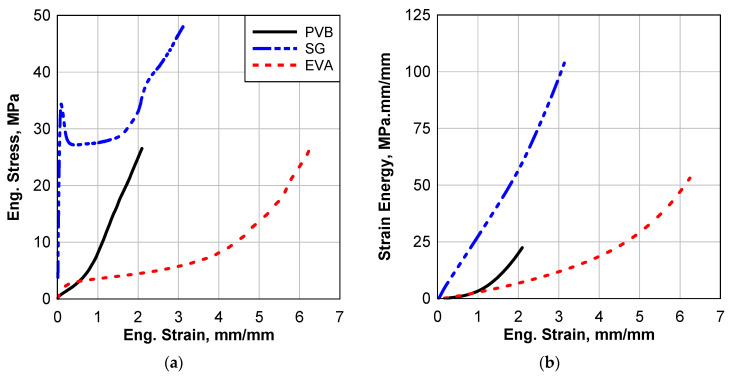
Static response of the control samples of cured interlayer materials; (**a**) stress–strain curve and (**b**) strain energy.

The water immersion environmental effect was induced by submerging the samples in distilled water for approximately one week at room temperature. During this period, the amount of water absorption for each material was recorded. After the immersion, the samples were subsequently dried in an oven and subjected to testing at room temperature. This section will present and analyze the outcomes of the cured/processed materials both with and without the water immersion environmental effect. All materials underwent static tests, and Figure 6 illustrates the static results of all the materials without the presence of environmental effects.

As outlined in Table 3, The strength of PVB, SG, and EVA interlayers are 26.53, 48.05, and 26.31 MPa, respectively, and the failure strains are 2.096, 3.14, and 6.24, respectively. SG5000 has the highest strength while EVA has the highest failure strain, as seen in Figure 6a. Although static testing was performed, the strain energy, shown in Figure 6b, indicates the performance of these materials under dynamic loads. It can be seen that SG can absorb the maximum amount of strain energy of 103 MPa.mm/mm before the final failure, and PVB absorbs the minimum amount. EVA is the weakest material in terms of strength, but it can absorb energy 236% higher than PVB due to its higher failure strength.

The stress–strain relation of PVB is a typical hyperplastic exponential relation, although there are small initial linear and plastic hardening parts for strains less than 0.05 mm/mm. EVA is similar to PVB except that the linear and hardening portion of the curve occurs at a strain less than 0.45 mm/mm. The yield strength of PVB and EVA is 0.3 and 2 MPa, respectively. After these strain values, the relation increases exponentially up to failure.

For the SG5000, the stress–strain curve is completely different. The curve started with a linear part with a very sharp slope achieving about 70% of its maximum strength before it drops suddenly to 56% of its maximum strength at a strain of 0.094 mm/mm. After that, by increasing the strain, the stress remains almost constant until a stain of 1.8 mm/mm. The stress increased exponentially after that point up to failure. It is worth noting that at 0.094 strain, the deformation of the samples localizes at a specific zone. 

**Figure 7:**
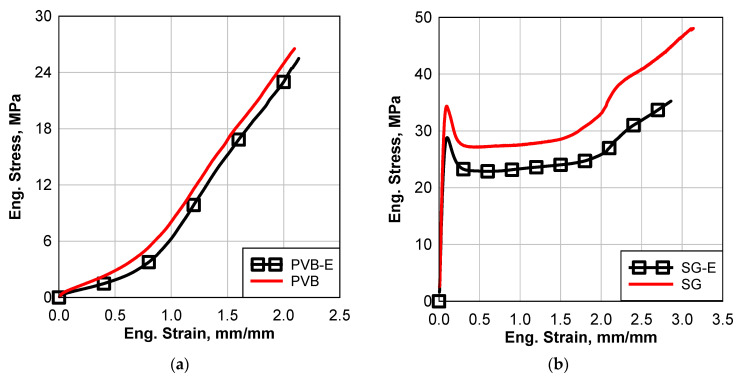

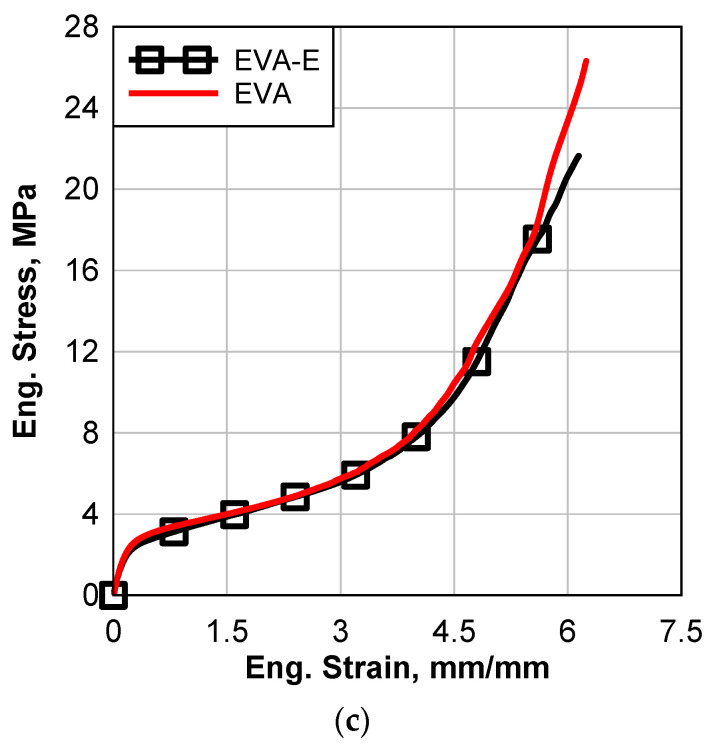
Comparison between stress–strain results of all the materials with and without environmental effect; (**a**) Saflex Standard Clear PVB, (**b**) Kuraray SG5000, and (**c**) EVA EVGARD.

Figure 7 shows a comparison between the interlayer materials with and without the environmental effects; adding the letter E to the end of the material name means that it was subjected to the water immersion effect. The water immersion environmental effect caused degradation of all the materials, as shown in Figure 7. The degradation causes a reduction in the stiffness and strength of all the materials. At a strain rate of less than 5.5 mm/mm, this degradation is more significant in the case of PVB and SG5000, as shown in Figure 7a,b, which caused the strength to be reduced by 4% and 17.75%. The water immersion effect had a minor impact on the EVA material, as depicted in Figure 7c. However, as the strain rate exceeded 5.5 mm/mm, the difference became more pronounced. In contrast, the SG5000 material experienced a significant reduction in failure strain due to water immersion, while the strains of PVB and EVA were only slightly affected. In conclusion, the water immersion environmental effect led to a softening of all materials, with the softening being most pronounced in the case of SG5000 and least in the case of EVA. Nevertheless, for large strain rates (greater than 5.5), the EVA material underwent significant alterations.

**Figure 8:**
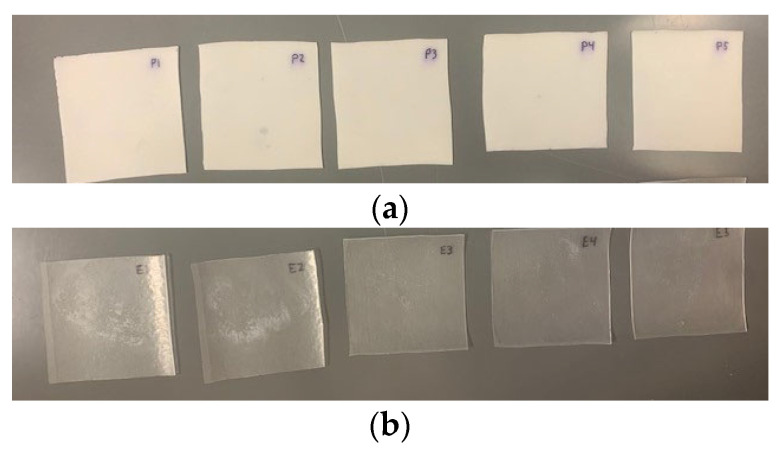
Immersed PVB versus EVA visual comparison; (**a**) immersed PVB and (**b**) immersed EVA.

Regarding the physical properties, the PVB specimens exhibited opaqueness when immersed in water, displaying a milky-white appearance instead of their usual clarity (Figure 8a). However, this opaqueness disappeared after the specimens were dried in the oven at the end of the 168-hour weighing period. Such a change in appearance could potentially pose visibility challenges for LG windows bonded with PVB, mainly due to humidity migration effects. On the other hand, the opaqueness of EVA was not significantly affected by water immersion, as illustrated in Figure 8b, which helps explain the lesser impact on the material’s mechanical properties.

**Figure 9:**
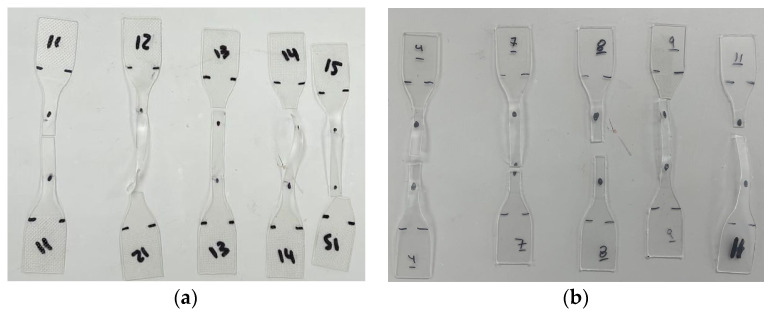
Specimens after testing: (**a**) PVB and (**b**) SG.

The failure modes observed for the three materials displayed distinct characteristics, as indicated in Figure 9. In the case of PVB, the deformation appeared to be uniformly distributed over the sample until it was cut at the middle of the gauge length, see Figure 9a. Conversely, for EVA, the sample exhibited much greater stretching compared to PVB and SG5000. This excessive strain caused the straight area of the specimen to turn white, followed by its eventual breakage. As for SG5000, after the initial peak or yielding, there was a sudden reduction in stress accompanied by strain localization in the middle of the sample, see Figure 9b. This localized strain persisted until the end of the test. This reduction in stress after the yield stress might be attributed to surface cracking, as the surface of SG5000 appeared brittle in contrast to EVA and PVB.

### 3.2. Diffusion Coefficient of Water in Polymers

**Figure 10:**
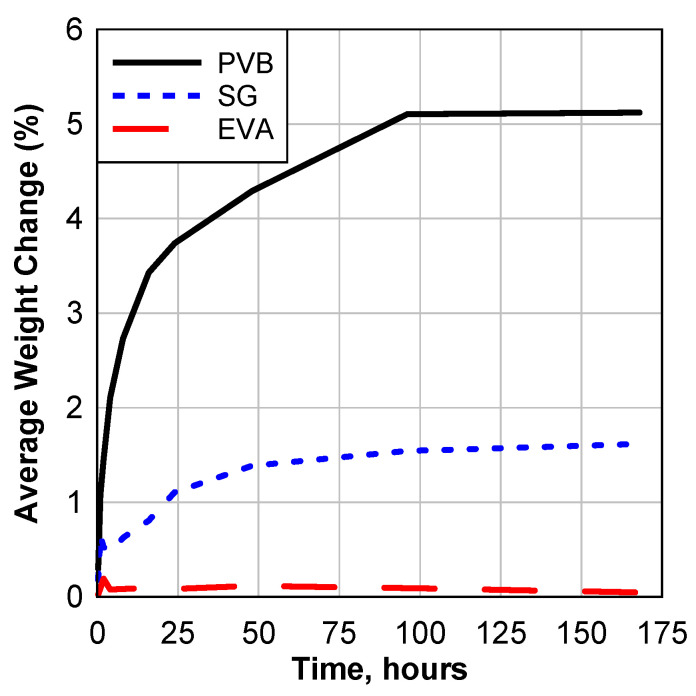
Water content over time.

Figure 10 shows the average weight change due to the water immersion during the week. PVB has the maximum absorption, followed by SG5000. EVA did not absorb much water, which explains why its performance was not significantly affected by this environmental effect. The maximum weight gain percentages were 5.1, 1.6, and 0.2% for PVB, SG5000, and EVA.

PVB absorbs water rapidly, reaching equilibrium around 100 h, making it less ideal for high-moisture environments. EVA, designed for minimal moisture absorption, absorbs water more slowly and reaches a lower equilibrium level than PVB and SG. SG, balancing between PVB and EVA, absorbs moisture faster than EVA but slower than PVB, with a moderate equilibrium level. The choice among these interlayers depends on the application’s moisture exposure and performance requirements. Ogawa et al. presented a formula to calculate the diffusion coefficient (D) [47], as follows:

$$D=\frac{\pi }{16}I^{2}d^{2}$$

where d represents half the interlayer thickness, I is the gradient of the initial slope in the relation between $\frac{M_{t}}{M_{\infty }}$ and $\sqrt{t}$, $M_{t}$ is the mass of water absorbed by the material at time t, and $M_{\infty }$ is the mass of water absorbed by the material at equilibrium (i.e., the maximum absorption). Table 4 demonstrates the diffusion coefficients for PVB, SG, and EVA.

### 3.3. Bond/Shear Test Results

**Figure 11:**
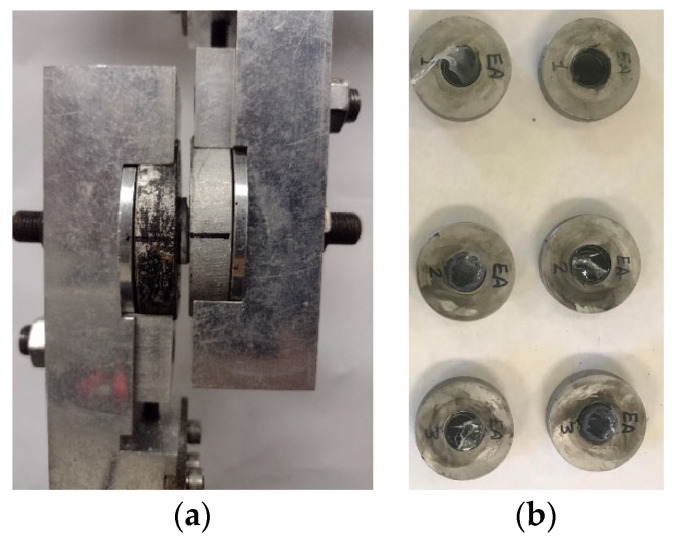
Bond test of laminated glass sample; (**a**) deformed shape during testing and (**b**) failed samples.

The laminated glass disk water immersion environmental effect was performed by immersing the LG samples in distilled water for about one week. The amount of water absorption was recorded for all the materials. After that, the samples were dried in the oven and tested at room temperature. Details about this procedure are found in the test setup section. A total of 36 samples were tested, representing 12 unique testing cases. The disks’ diameter is 19.0 mm (0.75 in), and all laminates have 6.3 mm (0.25 in) thick glass layers and 1.5 mm (0.06 in) thick polymer. Figure 11a shows the deformed shape of the sample during the testing, and Figure 11b shows some samples after the failure. It can be seen that all the samples failed at the interlayer. Stiff materials such as SG samples failed due to the peeling of the interlayer from the glass surface, while soft materials such as EVA failed due to the tearing of the polymer interlayer. The differential displacement between the two disks is monitored by the high-resolution camera using the black marks on the edge of the aluminum enclosure. Load data are evaluated from the machine load cell and the displacement data are evaluated from the image-processing software (Phototrack V2021). The load is then converted to shear stress by dividing it by the area of the disks (π/4 (19 mm)^2^). The shear strain is evaluated by dividing the differential displacement of the aluminum encloser by the interlayer thickness, i.e., 1.5 mm (0.06 in). Every laminate was tested in a dry case (DS), saturated case (SS), and saturated dry case (SDS). Three samples were tested for every case, and the average stress–strain results are calculated.

**Figure 12:**
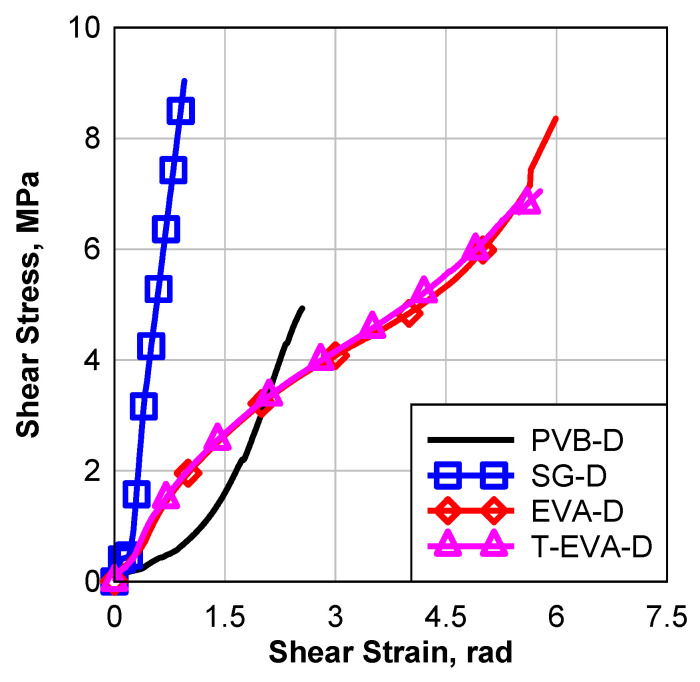
Bond test of laminated glass control group (D).

Before discussing the environmental effect, a comparison will be made between the stress–strain curves of the four laminates without the effects, i.e., D cases, as seen in Figure 12. It can be observed that the performance of PVB is a hyperplastic performance; however, both the EVA laminates have three stages: a linear part, a hardening part, and a hyperelastic experimental part similar to the performance of PVB. The SG laminate has an almost linear part up to failure. EVA and SG performance is very similar to the uniaxial tension samples’ performance; however, SG is different due to the absence of the post-yielding softening part. 

The result summary of all the testing groups is listed in Table 5. Comparing the D cases to the S cases shows that the water saturation caused a significant strength reduction for all the materials. For SG samples, the interlayer was deboned during the mounting of the sample to the testing fixture, which means that the laminate completely lost its bonding strength. However, PVB strength was reduced by 73% after saturation for one week. For EVA with annealed glass, the sample strength was reduced by 21%; however, the tempered glass sample strength was not affected by the water immersion.

Upon drying the samples again, i.e., SD cases, they recovered some of their strength. Compared to the control sample, i.e., D cases, the PVB and SG samples reduced by 41% and 4.4%, respectively, after removing the saturation effect. The interesting finding is that although SG completely lost its strength when tested as saturated, it gained all its strength when dried again. For EVA, after saturation and drying cycle, its strength was reduced by 28% for the case of annealed glass and increased by 12% for the case of tempered glass. For the failure strain, the saturation caused it to reduce by 27, 14, and 0.34% for PVB, EVA with annealed glass, and EVA with tempered glass, respectively. The full saturation and drying cycle, i.e., SD cases, caused a change of −21, −1, −17.1, and +5.35% of the dry control samples strength of PVB, SG, EVA, and T-EVA, respectively. 

**Figure 13:**
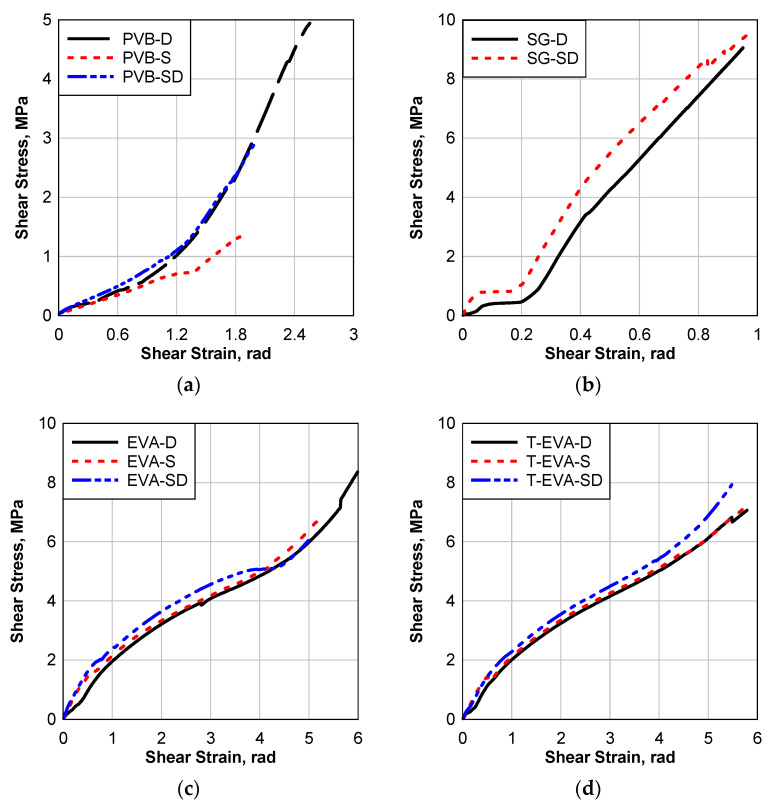
Static stress–strain curve of bond testing with and without environmental effects; (**a**) PVB laminate, (**b**) SG5000 laminate, (**c**) EVA laminate, and (**d**) T-EVA laminate.

Figure 13 shows the stress–strain relations of the four laminates used in this study. From Figure 13a, it can be seen that there was a significant softening in the stress–strain curve of PVB due to the saturation and reduction in both the failure stress and strain as mentioned before. After drying, the SD stress–strain curve almost coincided with the D curve; however, it was not able to recover the D case maximum stress and strain limits. Figure 13b shows the stress–strain relations of the SG laminates for D and SD cases. It can be seen that the SD curve is slightly stiffer than the D curve; in addition, SG laminate slightly achieved higher failure stress and strain, although it completely lost its strength when tested in the saturated case. This means that the saturated dry cycle enhanced the overall performance of the SG laminate. 

For the EVA and T-EVA laminate, comparing the dry case D to the saturated case S, it can be seen that the curves almost coincide, which gives EVA laminates a high advantage over the other two laminates. Both the S and SD cases showed a small enhancement in the resistance compared to the D case, with no big difference between the three cases. In addition, the strength of EVA laminates was slightly affected and reduced due to saturation; however, the strength of T-EVA was not affected. For SD cases, the resistance increased for both EVA and T-EVA laminates with more reduction in strength of EVA than T-EVA laminates compared to the control case D. Based on the previous discussion, it can be concluded that the EVA laminate showed the most stable behavior for the underwater immersion environmental effect. SG5000 showed the worst performance under the saturated case. 

## 4. Conclusions

This paper presents an experimental study that aims to investigate the impact of accelerated humidity on polymer interlayer materials and laminated glass panels. Uniaxial tension tests were conducted on the polymeric interlayer with and without exposure to water immersion. Additionally, bond tests were performed on laminated glass panels under the same conditions. The results include discussions on failure modes, maximum failure stress, as well as stress–strain curves and strain values. The following conclusions can be drawn from this study.

1. SG 5000 exhibited superior strength, surpassing PVB and EVA by 82% and 81%, respectively. However, EVA showed a higher failure strain, outperforming PVB and SG 5000 by 198% and 99%. Additionally, SG 5000 displayed a unique stress–strain curve with sudden drops and localized strain at specific points, indicating potential surface cracking.
2. Maximum-strain energy absorption (103 MPa.mm/mm) before failure was observed in SG, with PVB showing the least absorption. EVA, despite its lower strength, absorbed 236% more energy than PVB due to its higher failure strength.
3. EVA exhibited distinct stages in its stress–strain curve, including linear, nonlinear hardening, and hyperplastic stages, showcasing its complex behavior under stress.
4. Softening occurred in all materials due to water immersion, with SG5000 experiencing the most significant softening and EVA undergoing significant alterations at higher strain rates.
5. PVB exhibited the highest water absorption (5.1%), followed by SG5000 (1.6%), while EVA absorbed minimal water (0.2%).
6. Stiff materials like SG samples failed due to interlayer peeling, whereas soft materials like EVA failed due to polymer interlayer tearing.
7. SG samples lost all bonding strength after water immersion but regained it after drying, while PVB’s strength reduced by 73%. EVA’s strength varied post-immersion depending on the type of glass used.
8. EVA laminates showed exceptional stability and resilience under water immersion and the saturation–drying cycle, maintaining their integrity in various environmental conditions. In contrast, SG5000 exhibited the poorest performance when saturated, indicating high susceptibility to environmental effects.

These findings provide valuable insights into the behavior of polymer interlayers and laminated glass panels under accelerated humidity conditions, paving the way for improved materials and design considerations in real-world applications.

## Figures and Tables

**Table 1:** Polymer sheet used in this study.

Material	Manufacturer/Product	Thickness mm (in)	Material State
**PVB**	Estman, Kingsport, TN, USA/Saflex/Standard Clear	0.76 (0.03)	Virgin
**PVB**	Estman, Kingsport, TN, USA/Saflex/Standard Clear	1.52 (0.06)	Cured/processed
**EVA**	Salem Fabrication Technologies, Winston-Salem, NC, USA/EVGuard	0.76 (0.03)	Virgin
**EVA**	Salem Fabrication Technologies, Winston-Salem, NC, USA/EVGuard	1.52 (0.06)	Cured/processed
**SG5000**	Kurary, Houston, TX, USA/Kurary/SG5000	0.89 (0.035)	Virgin and cured

**Table 2:** Shear test disk samples.

Laminate	Layup	Manufacturer	Glass	Interlayer	# Samples
**PVB**	0.25A-0.06PVB-0.25A	OLDCASTLE, Orlando, FL, USA	Annealed	PVB	3 × 3
**SG**	0.25A-0.06SG-0.25A			SG	3 × 3
**EVA**	0.25A-0.06EVA-0.25A	MOAG, Georgetown, IN, USA	Annealed	EVA	3 × 3
**T-EVA**	0.25T-0.06EVA-0.25T		Tempered		3 × 3

**Table 3:** Summary of tensile test results.

Interlayer	# Samples	Max. Average Tensile Stress (MPa)	COV%	Max. Average Tensile Strain
PVB	5	26.53	1.21	2.096
SG	5	48.05	1.83	3.14
EVA	5	26.31	2.76	6.24
PVB-E	5	25.48	3.23	2.067
SG-E	5	35.24	2.83	2.9
EVA-E	5	21.64	2.24	6.14

**Table 4:** Diffusion coefficients.

Interlayer	I (s^−1/2^)	D
PVB	0.003	1.03
SG	0.002	0.45
EVA	0.0012	0.05

**Table 5:** Summary of bond/shear test results.

Test Summary	# Samples	Max. Average Shear Stress (MPa)	COV%	Max. Average Shear Strain
PVB-D	3	4.93	1.18	2.55
PVB-S	3	1.33	1.37	1.84
PVB-SD	3	2.92	1.13	2.01
SG-DS	3	9.05	1.89	0.95
SG-SS	3	-	-	-
SG-SS	3	9.45	1.93	0.96
EVA-D	3	8.44	2.42	6.02
EVA-S	3	6.67	2.63	5.16
EVA-SD	3	6.05	2.57	4.99
T-EVA-D	3	7.07	2.53	5.79
T-EVA-S	3	7.21	2.71	5.77
T-EVA-SD	3	7.94	2.59	5.48

## Data Availability

Data are contained within the article.
